# Multiple Chlamydiaceae Species in Trachoma: Implications for Disease Pathogenesis and Control

**DOI:** 10.1371/journal.pmed.0050014

**Published:** 2008-01-03

**Authors:** Deborah Dean, Ram P Kandel, Him K Adhikari, Tracey Hessel

**Affiliations:** 1 Center for Immunobiology and Vaccine Development, Children's Hospital Oakland Research Institute, Oakland, California, United States of America; 2 Department of Medicine, University of California at San Francisco, California, United States of America; 3 Joint Graduate Group in Bioengineering, University of California, Berkeley, and University of California, San Francisco, California, United States of America; 4 Department of Ophthalmology, Lumbini Rana-Ambika Eye Hospital, Bhairahawa, Nepal; 5 Department of Pediatrics, University of California at San Francisco, California, United States of America; National Center for Infectious Disease, United States of America

## Abstract

**Background:**

Chlamydia trachomatis is a unique obligate intracellular bacterium that remains the leading cause of sexually transmitted bacterial diseases and preventable blindness worldwide. Chronic ocular infections are referred to as trachoma, and predominate in developing countries. Since 2001, the World Health Organization has promoted control strategies including antibiotics, improved hygiene, and environmental measures with limited success. Consequently, a vaccine is urgently needed. Integral to vaccine design is an understanding of the interactions of the pathogen and host immune response. Various animal models of trachoma show that urogenital C. trachomatis strains and other species of the family Chlamydiaceae produce severe conjunctival inflammation and scarring similar to that of the ocular C. trachomatis strains. However, we do not know the extent of organisms that may be involved in human trachoma. Furthermore, C. trachomatis heat shock protein 60 (Hsp60) has been implicated in inflammation and conjunctival scarring but the role of other Chlamydiaceae Hsp60 in disease pathogenesis has not been examined. In this study, we set out to identify whether other Chlamydiaceae species are present in trachoma, and determine their association with severity of clinical disease and with mucosal and systemic immune responses to Chlamydiaceae species-specific Hsp60 to further investigate the immunopathogenesis of this blinding disease.

**Methods and Findings:**

We randomly selected nine of 49 households in a trachoma-endemic region of Nepal. Trachoma was graded, and real-time, quantitative (k)PCR was used to detect genomic DNA and cDNA (from RNA) for Chlamydiaceae *ompA* and 16S rRNA genes, respectively, from conjunctival swabs. IgG antibody responses to recombinant (r) Chlamydiaceae species-specific Hsp60 were determined for tears and sera. Surprisingly, all three species—*C. trachomatis, Chlamydophila psittaci,* and Chlamydophila pneumoniae—were detected in eight (89%) study households; one household had no members infected with C. pneumoniae. Of 80 (63%; *n* = 127) infected individuals, 28 (35%) had infection with C. psittaci, or *C. pneumoniae,* or both; single and dual infections with C. psittaci and C. pneumoniae were significantly associated with severe conjunctival inflammation (OR 4.25 [95% confidence interval (CI), 2.9–11.3], *p* = 0.009] as were single infections with C. trachomatis (OR 5.7 [95% CI, 3.8–10.1], *p* = 0.002). Of the 80 infected individuals, 75 (93.8%) were also positive for 16S rRNA by kPCR for the same organism identified by *ompA*. Individuals with tear IgG immunoreactivity to Chlamydiaceae rHsp60 were eight times more likely than individuals without tear immunoreactivity to be infected (95% CI 6.4–15.1; *p* = 0.003), 6.2 times more likely to have severe inflammation (95% CI 4.4–12.6; *p* = 0.001), and 5.7 times more likely to have scarring (95% CI 3.9–11.1; *p* = 0.019) while individuals with serum IgG immunoreactivity were 4.1 times more likely to be infected (95% CI 3.1–10.1; *p* = 0.014).

**Conclusions:**

We provide substantial evidence for the involvement of C. psittaci and *C. pneumoniae,* in addition to *C. trachomatis,* in trachoma. The distribution of Chlamydiaceae species by household and age suggests that these infections are widespread and not just sporadic occurrences. Infection with multiple species may explain the failure to detect chlamydiae among active trachoma cases, when only C. trachomatis is assayed for, and the failure of clinically active cases to resolve their disease following what would be considered effective C. trachomatis treatment. The evidence for viable (RNA-positive) organisms of all three species in single and coinfections, the significant association of these infections with severe inflammation, and the significant association of tear and serum IgG responses to Chlamydiaceae Hsp60 with inflammation and scarring, support the role of all three species in disease pathogenesis. Thus, while our findings should be confirmed in other trachoma-endemic countries, our data suggest that a reevaluation of treatment regimens and vaccine design may be required. Understanding the full impact of Chlamydiaceae species on the epidemiology, immunopathology, and disease outcome of trachoma presents a new challenge for Chlamydiaceae research.

## Abstract 2

In a study of trachoma cases within households in Nepal, Deborah Dean and colleagues find involvement of the *Chlamydia* species *C. psittaci* and *C. pneumoniae* in addition to *C. trachomatis*.

## Editors' Summary

**Background.:**

Six million people—most of whom live in crowded, unhygienic conditions in developing countries—are blind because of an infectious disease called trachoma. It is generally accepted that trachoma is caused by Chlamydia trachomatis, bacteria that pass easily between people on hands and clothing. Infection usually occurs first during childhood, but people do not become blind until adulthood. Successive infections cause progressive scarring of the inside of the eyelid. Eventually, the eyelashes turn inward and rub painfully over the front of the eye (the cornea). This causes corneal scarring, loss of corneal transparency and, finally, irreversible blindness. C. trachomatis infections can be prevented by improving personal hygiene (in particular, facial cleanliness in children) and by reducing fly breeding sites, and they can be treated with antibiotics. However, C. trachomatis and other organisms appear to be developing drug resistance to antibiotics commonly used to treat these infections. In addition, early scarring and in-turned eyelashes can be treated surgically, although recurrence of the in-turned eyelashes frequently occurs months to years after surgery.

**Why Was This Study Done?:**

The World Health Organization has been promoting these “SAFE” interventions (surgery, antibiotics, facial cleanliness, and environmental improvement) since 2001 with the aim of eliminating trachoma by 2020. However, these control measures have had limited success so far and it looks like a vaccine may also be needed. To develop an effective vaccine, scientists need to know whether all cases of human trachoma are caused by so-called ocular strains of C. trachomatis. Might C. trachomatis strains that are usually associated with sexually transmitted disease (urogenital strains) or different species in the family Chlamydiaceae also cause human trachoma as work in animals has suggested? In this study, the researchers have investigated which Chlamydiaceae species are associated with trachoma in a region of Nepal where the disease is endemic (always present).

**What Did the Researchers Do and Find?:**

The researchers examined all the members for trachoma in nine randomly selected households in a Nepali village. They then used sensitive molecular biology methods to identify the species in the family Chlamydiaceae and strains present in the eyes of the infected individuals. One third of them were infected with only C. trachomatis (mainly ocular strains but also some urogenital strains), one in five were infected with only Chlamydophila psittaci, and one in ten with only Chlamydophila pneumoniae. The other infected individuals had mixed infections. Infection with C. psittaci and/or C. pneumoniae was strongly associated with severe eye inflammation as was infection with C. trachomatis alone. The researchers also asked whether there were any antibodies (proteins made by the immune system that recognize infectious organisms) in the tears or blood of the infected individuals that recognized the Hsp60 protein of each Chlamydiaceae species; an immune response to C. trachomatis Hsp60 is thought to be involved in the inflammation and scarring seen in trachoma. Individuals with antibodies in their tears to Chlamydiaceae Hsp60, the researchers report, were eight times as likely to be actively infected with these bacteria and six times as likely to have severe eye inflammation as individuals without the antibodies.

**What Do These Findings Mean?:**

These findings provide evidence for the widespread involvement of C. psittaci, *C. pneumoniae,* and urogenital strains of C. trachomatis as well as ocular strains of C. trachomatis in trachoma and might explain why some people with active trachoma do not have C. trachomatis in their eye secretions and why antibiotics that kill C. trachomatis effectively do not cure all cases of trachoma. However, because live bacteria were not isolated from patients and shown to cause disease in a model system, these findings do not prove that Chlamydiaceae other than C. trachomatis cause trachoma. Some or all of the bacterial strains and species detected in this study may be innocent bystanders although the strong association between their presence and severe inflammation and the association between antibody responses to Chlamydiaceae Hsp60 and inflammation argues against this possibility. If the involvement of multiple Chlamydiaceae strains and species is confirmed and extended in other trachoma-endemic regions, then future antimicrobial therapies and vaccines will need to deal with all these bacteria and not just C. trachomatis.

**Additional Information.:**

Please access these Web sites via the online version of this summary at http://dx.doi.org/10.1371/journal.pmed.0050014.

- The MedlinePlus encyclopedia contains a page on trachoma (in English and Spanish)
- The World Health Organization provides information on trachoma (mainly in English but some information is available in French, Russian, and Spanish)
- The US Centers for Disease Control and Prevention provides a technical fact sheet on trachoma
- The charity Sightsavers International also provides information on trachoma and global efforts to eliminate the disease
- The Carter Center provides an overview of trachoma control and a description of its trachoma control program

## Introduction

Chlamydia trachomatis is a unique obligate intracellular bacterium that is the leading cause of bacterial sexually transmitted and blinding diseases worldwide. Trachoma is a chronic disease of the conjunctival mucosa that can lead to blindness 10–40 y after infection, and is found disproportionately among resource-poor nations [1]. Thus, trachoma has a significant global impact on agrarian economies [2]. The estimated number of people with trachoma who will develop blindness by the year 2020 is 12 million [3], which has placed trachoma on the World Health Organization's (WHO) priority list for intervention [4].

While a number of trachoma-endemic countries have implemented the SAFE intervention strategy to prevent blinding trachoma (S, surgery for trichiasis, defined as ≥1 eyelash touching the globe of the eye; A, oral antibiotics to treat C. trachomatis; F, facial cleanliness; and E, environmental improvement), often only the S and/or A components are adopted. Consequently, success rates are low, and infection and disease often reemerge. Postoperative rates of trichiasis recurrence are high even with treatment for C. trachomatis at the time of surgery [5–8], and C. trachomatis infection rates return within a year or two following cessation of mass or targeted antibiotic treatment programs [9–14]. The latter may in part be due to an accelerated rate of reinfection following azithromycin treatment, which may blunt the immune response to the organism and lead to a population with increased susceptibility to infection [14–16]. Consequently, there remains a gap in our knowledge regarding transmission, recurrence, and persistence of infection and the immunopathogenesis of trachomatous disease.

The predominant C. trachomatis serological variants (serovars) considered responsible for trachoma are A, B, Ba, and C, which are distinguished on the basis of antibody typing of the major outer membrane protein (MOMP) [17] or genotyping of *ompA*, the gene that encodes MOMP. Additional *ompA* genotypic subtypes of these serovars have been identified in trachoma-endemic regions of Asia and Africa [18–20].

From animal models of trachoma, there is some evidence to suggest that nontrachoma serovars may be important in disease pathogenesis. Early studies in Formosan rock macaques (Macaca cyclopis) showed that conjunctival infection with trachoma serovars A, B, or C was associated with pannus formation in only 0.5% of previously infected animals compared with 12% of animals infected with serovars D, E, and F and subsequently challenged with the homologous serovar [21]. Pannus is a common ocular finding in trachoma, occurring as neovascularization of the cornea. The latter serovars typically infect the urogenital mucosa, but can also cause follicular conjunctivitis and punctate keratitis [22–25]. In another study of cynomolgus monkeys (M. fascicularis), serovar E produced more severe ocular disease in primary and secondary challenge experiments compared with serovars B or C [26]. These findings were similar to those of Wang et al. [21], where serovar E induced a more severe conjunctival inflammatory response in M. cyclopis than serovar C. Indeed, serovars D, E, G and F have been isolated from trachoma populations in Asia and Africa [27]. In one study by Harrison et al. [28], serovar J was isolated from the conjunctivae of a Navajo child with clinical trachoma, suggesting that there is more disease overlap of urogenital strains with trachoma than previously recognized. Similarly, urogenital strains were identified among 14 Danish patients in Denmark who had clinical trachoma, five of whom had severe disease [29]. These cumulative data suggest that sexually transmitted serovars may be overlooked in disease pathogenesis in trachoma-endemic regions. However, the precise association between C. trachomatis sexually transmitted disease strains and trachoma remains to be determined. Furthermore, the possibility of ocular infections caused by *Chlamydophila* species has not been considered in trachoma-endemic areas.

C. psittaci and *C. pneumoniae* have been implicated in conjunctivitis in rare instances involving zoonotic or laboratory transmission [30–32]. The infrequent involvement of other Chlamydiaceae species is most likely due to the fact that these species are not considered in the etiology of ocular disease and, therefore, are not assayed for. However, Chlamydophila caviae (previously referred to as Chlamydia psittaci, strain GPIC) and Chlamydophila felis (previously Chlamydia psittaci, strain Fe/C-56) are known to be important ocular pathogens in guinea pigs and cats, respectively. Both cause an inflammatory response similar to that of trachoma [33–35]. Conjunctivitis in swine has also been associated with Chlamydia suis [36]. More importantly, the field of Chlamydiaceae research has recently experienced an explosion in the discovery of new organisms in the order Chlamydiales isolated from amphibian, mammalian, and environmental sources [37]. These findings suggest the need for a more in-depth evaluation of Chlamydiales organisms that may be present in trachoma-endemic regions and that may play a role in ocular disease. If non-*C**.** trachomatis* species are also involved, this would dramatically change the current approach to the study and treatment of trachoma.

The objective of this study was to identify Chlamydiaceae species responsible for trachoma in an endemic region of Nepal. We also explored the association of single and coinfections with Chlamydiaceae species with severity of clinical disease, and with mucosal and systemic immune responses to Chlamydiaceae species-specific heat shock protein 60 (Hsp60) to provide further information on the immunopathogenesis of this blinding disease.

## Methods

### Ethical Approval

Institutional Review Board approval was obtained from the Nepal Netra Jhoti Shang, Kathmandu, Nepal, and Children's Hospital Oakland Research Institute, Oakland, California, United States.

### Study Population and Specimen Collection

The study population comprised individuals from a trachoma-endemic village of the Lumbini Zone in Southwestern Nepal. All 146 individuals over 6 mo of age residing in nine (18%) randomly selected households of 49 households in the village were included in the study after they gave informed consent. A table of random numbers was used for selection. Sera were collected, and tears were obtained by applying Weck-cel sponges (Edward Weck, Research Triangle Park, North Carolina, United States) to the inner canthus of each eye and allowing sponges to reach saturation. Conjunctival scrapings of the bilateral upper tarsal conjunctivae were obtained using Dacron swabs (Hycor Biomedical, Portland, Maine); those performing the scrapings wore gloves that were changed between individuals to avoid cross contamination. The swabs were immediately placed in ∼ 1 ml of SPG chlamydiae collection media in an RNase- and DNase-free cryovial as described previously [38]. Another swab of the right eye was obtained that alternated with the swab for SPG, and was placed in M4 transport media (Remel, Lenexa, Kansas, United States). All samples were immediately frozen in liquid nitrogen for transport to the laboratory where they were stored in liquid nitrogen until use.

### Trachoma Grading

Both upper eyelids of study participants were examined for trachoma and photographed. Each was graded using the modified WHO trachoma grading scale of Thylefors et al. [39]. Briefly, T0, absence of trachoma, was defined as < 5 follicles on the lower 2/3 of the upper tarsus; TF, follicular trachomatous inflammation, as ≥ 5 follicles; TI, intense trachomatous inflammation, as > 50% of the upper tarsal blood vessels obscured by inflammation; TS as trachomatous conjunctival scarring; and TT, trachomatous trichiasis, as ≥ 1 lash touching the eye globe. Active trachoma was defined by the presence of either TF or TI or both. A final grade required consensus among two of three independent readers (DD, TH, and RPK) who were kept unaware of demographic and microbiologic results.

### Screening for Chlamydiaceae Species Using Genomic DNA and 16S rRNA by Real-Time Quantitative PCR

To avoid cross-contamination in the laboratory, different rooms were used for sample preparation, for set-up of PCR or kPCR, for sample runs in the thermocycler for PCR or the ABI7000 instrument for kPCR, and for agarose gel electrophoresis for PCR products that were subsequently used for DNA purification and sequencing. In addition, the BSL2 biosafety hood used for sample processing in the first room was decontaminated with 10% bleach and with 30 min of exposure to UV light between use. Personnel changed gloves between batches of samples processed (*n* = 9 + 1 negative control) and between rooms. Lab coats designated for a specific room were used only in that room.

M4 samples were processed according to the Amplicor PCR test (Roche Diagnostics, Indianapolis, Indiana, United States) package insert using previously described techniques [5] to detect the presence of C. trachomatis using appropriate kit negative and positive samples.

Genomic DNA and RNA were purified according to manufacturer's instructions (DNA and RNeasy kits, Qiagen, Valencia, California, United States) and as we previously described [40] from two separate aliquots of 200 μl of SPG sample. RNA was reverse transcribed to cDNA using TaqMan RT reagents (Applied Biosystems, Foster City, California, United States), as described [40]. Primers were designed using Primer Express (Applied Biosystems) to amplify eukaryotic *β-actin*, species-specific *ompA* (DNA) for C. trachomatis, *C. pneumoniae,* and *C. psittaci,* the 16S rRNA (cDNA from RNA) for known species of the two genera of Chlamydiaceae, *Chlamydia* and *Chlamydophila*, and the 19 reference strains of *C. trachomatis,* and species-specific 16S rRNA (cDNA) for *C. trachomatis, C. pneumoniae,* and C. psittaci using either sequences of each from GenBank that were aligned in Megalign (DNASTAR) as previously described [41,42] or sequences that were previously published [41–45] (Table 1). Primers were subjected to a BLAST search to ensure that they did not match any published human or other microbial organism genes.

**Table 1:**
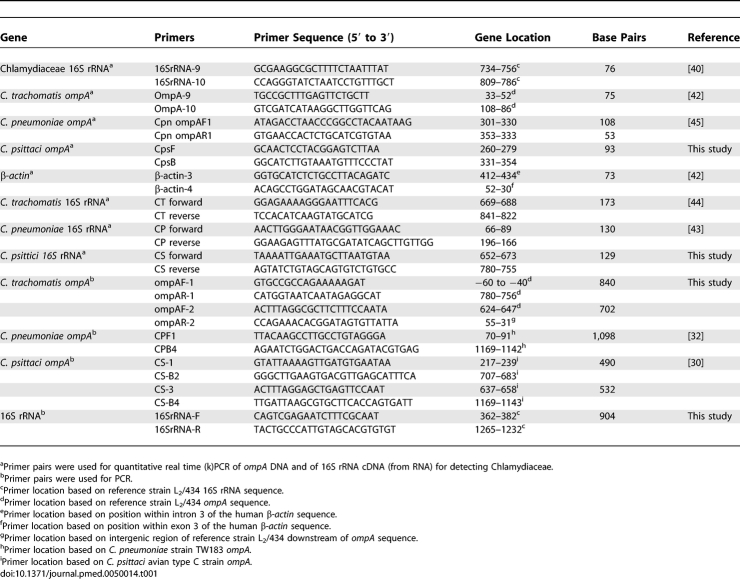
Oligonucleotide Primers used for Quantitative Real-Time (k)PCR and PCR

For real time quantitative (k)PCR, we used our existing clones of the single-copy *ompA* gene of *C. trachomatis,* Chlamydiaceae-specific 16S rRNA and eukaryotic *β-actin* gene, and we generated additional gene clones as previously described [41,42], using the TOPO-TA cloning kit (Invitrogen, Carlsbad, California, United States) and PCR products from the above-stated primers, for the *ompA* gene for both C. pneumoniae and C. psittaci, and for species-specific regions of 16S rRNA for all three species. Standards were made by using 16 serial 2-fold dilutions (3.3 × 10^4^ to 1 copy of each clone) of each gene clone and were amplified in the same plate as the samples and controls. Since each gene clone represents a single-copy gene in the respective genome, this enabled us to generate standard curves for each gene analyzed and determine absolute quantities for each Chlamydiaceae species in each sample, as described previously [41,42]. Thus, each 96-well plate included duplicates of DNA and cDNA sample templates and the clones for amplification of the three genes and of the standard curves for 16S rRNA and *ompA* for each Chlamydiaceae species and for *β-actin* using the same cycling profile, SYBR mastermix, reagents, and negative controls, as we described previously [40]. The concentration of each gene was determined by comparison with the respective standard curve; *β-actin* was used to normalize *ompA* and 16S rRNA results [42]. kPCR for the detection of Chlamydiaceae species has also been successfully developed and employed by others [46,47].

### *ompA* and 16S rRNA Typing of Chlamydiaceae Species and Strains

Samples that were positive by the Amplicor test and/or kPCR for *ompA* and/or 16S rRNA underwent sequencing to identify each species and *ompA* genotype present in the templates. PCR primers for *ompA* and 16S rRNA (Table 1), and PCR volume, reagents, and positive and negative controls were used in each PCR run, and thermocycling and Big Dye Terminator automated capillary sequencing were performed, as we previously described [41]. Samples that had multiple peaks in a single nucleotide lane on the *ompA* and/or 16S rRNA electropherograms representing two or more genotypes or species were subjected to additional PCR using the respective species-specific primers for *ompA* (Table 1) [32,48] and sequenced as described above.

### Recombinant Fusion Proteins for Chlamydiaceae Species-Specific Hsp60 and Immunoblots for Anti-Chlamydiaceae Hsp60 Antibodies in Tears and Sera

We had previously reported a significant association of chlamydia-specific antibodies in tears to C. trachomatis-specific Hsp60 among individuals with trichiasis compared to controls [49]. Others have also shown an association of serum antibody responses to C. trachomatis Hsp60 with trachomatous scarring [50], a precursor to trichiasis. We constructed recombinant glutathione-*S*-transferase (GST) fusion proteins for C. psittaci-specific and C. pneumoniae-specific Hsp60 based on the respective sequences available in GenBank using techniques we have described [49]; we had previously constructed GST fusion proteins for C. trachomatis-specific Hsp60 and MOMP [49].

We assayed individual tears and sera with the recombinant *C. trachomatis, C. pneumoniae,* and *C. psittaci* Hsp60 (rCtHsp60, rCpnHsp60, and rCpsHsp60, respectively), all three combined Chlamydiaceae species-specific recombinant Hsp60 and recombinant MOMP (rMOMP) in triplicate using techniques as described previously [49]. Positive and negative control tears and sera were included in each blot. All assays were performed masked as to their infection status. Immunoblot results were determined using densitometry (Bio-Rad Gel Doc, BioRad, Hercules, California, United States). The minimum concentration for a positive result (immunoreactivity to rCHsp60, rCpnHsp60, or rCpsHsp60 or to all three recombinant Hsp60) was defined as the optical density of three standard deviations above the GST background density of the mean of nine negative control samples.

### Data Analysis

The outcome variables were trachoma grade, infection status (single or mixed Chlamydiaceae infections), infecting species and/or *ompA* genotype, and tear and serum antibodies to each Chlamydiaceae recombinant Hsp60 proteins and to all three recombinant Hsp60s. Active trachoma was defined as TF, TI, or TF and TI. Infection with Chlamydiaceae was defined as a positive Amplicor test and/or kPCR result for the *ompA* and/or 16S rRNA genes. The quantity of Chlamydiaceae was expressed as copy number per 100 eukaryotic cells (expressed as Chl/100) in the sample so as to normalize the data and ensure values >1, as we have described previously [42]. The species and *ompA* genotype were defined based on *ompA* and 16S rRNA gene sequences compared against all Chlamydiales and non-Chlamydiales sequences in GenBank using BLAST. Mixed infections were defined as those containing more than one *C. trachomatis ompA* genotype or more than one Chlamydiaceae species based on sequencing results of *ompA* and/or 16S rRNA as described above.

Associations between discrete variables were analyzed by Pearson Chi-square or Fisher exact test using Stata 9 (College Station, Texas, United States). Immunoreactivity to rCtHsp60, rCpnHsp60, rCpsHsp60 or to all three recombinant Hsp60 was reported as a negative or positive result (as the optical density of three standard deviations above the background density of the mean of nine negative control samples). These results were compared for individuals with evidence of any species infection versus uninfected individuals, and compared for clinical grade of trachoma, age, and sex. Multivariate analyses were performed for unconditional logistic regression. Possible confounding variables such as age and sex were included in the model, and weighted odds ratios were obtained after stratified analyses were performed. Household variables were included in the final model to correct for cluster sampling using the Monte Carlo method [51] as previously described [49].

## Results

### Demographics and Trachoma Grade

The study population comprised 146 individuals residing in nine households, and ranging in age from 1 to 87 years, with a mean age of 19 years. Sixteen (11%) of 146 individuals had T0, 40 (28%) had TF, 53 (36%) had TI, and 37 (25%) had TS; 20 (13.7%) of those with TS had TT. There was no significant difference by age as a continuous variable or gender for trachoma grade except that females tended to have a higher rate of TS and TT than did males**.** For all trachoma grades, the distribution of infection as determined by kPCR for *ompA* DNA and 16S rRNA cDNA was similar (see below).

### Chlamydiaceae Infections among the Study Population

Of a total of 127 individuals for whom conjunctival swabs were available, 80 (63%) individuals had chlamydial infection as determined by kPCR of DNA for Chlamydiaceae *ompA* and 75 (59%) had infection by kPCR of cDNA from RNA for Chlamydiaceae *16S rRNA*, while only 49 (38.6%) were positive by Amplicor PCR (Table 2). The quantitative values for kPCR were ≥ 10 Chlamydiaceae per 100 eukaryotic cells for *ompA* and ≥ 12 for 16S rRNA, which we defined as positive. The range was 10 to 3,500 Chlamydiaceae per 100 cells (mean, 352 per 100 cells) for *ompA* and 12 to 2,568 (mean, 289 per 100 cells) for 16S rRNA, and none fell below 10/100. There were three samples with no detectable *β-actin,* suggesting that the samples had no eukaryotic cells and were, thus, inadequate. These three samples were not used in the analyses. Table 2 shows the results for infection by trachoma grade. The quantitative range and mean per 100 eukaryotic cells for both *ompA* and 16S rRNA results by kPCR are provided in brackets for the results of kPCR to show the strength of the association with trachoma grade. Any sample with fewer than ten Chlamydiaceae per 100 eukaryotic cells was considered below the threshold for a positive sample. To show similarity, the Cohen kappa was used and agreements were 75.6% (kappa 0.54 [standard error (SE) = 0.079], 78% (kappa 0.58 [SE = 0.081]), 96% (kappa 0.92 [SE = 0.088]) for Amplicor PCR versus *ompA*, Amplicor PCR versus 16S rRNA, and *ompA* versus 16S rRNA, respectively. Corresponding *p*-values for each were < 0.001. Kappa value for all three tests was 0.662, with *p* < 0.001. All Amplicor- and 16S rRNA-positive samples were positive for the respective *ompA* gene of that species. Sixty-four (80%) of the infected individuals had infections in both eyes.

**Table 2:**
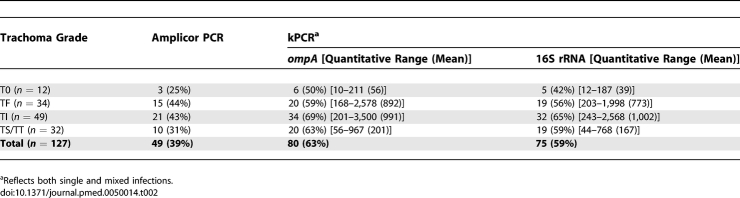
Correlation of Trachoma Grade with Amplicor PCR and Real-Time Quantitative (k)PCR of Chlamydiaceae *ompA* DNA and Chlamydiaceae 16S rRNA cDNA (from RNA)

**Figure 1:**
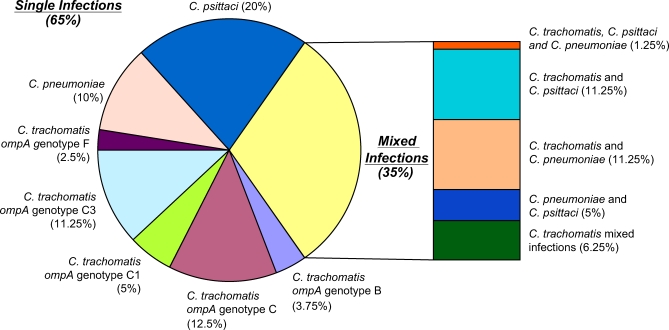
Chlamydiaceae Infections in a Trachoma-Endemic Region of Southwestern Nepal as Determined by 16S rRNA and *ompA* Genotyping Single infections included each species and the designated *ompA* genotypes. The percentages listed after each species or *ompA* genotype represent the number of individuals infected with that species or genotype divided by the total number of infected individuals with any species or *ompA* genotype (*n* = 80). Mixed infections included mixed *C. trachomatis ompA* genotypes, C. trachomatis genotype(s) with other Chlamydiaceae species, and coinfections with C. psittaci and C. pneumoniae.

Infections were further characterized by Chlamydiaceae species, *C. trachomatis ompA* genotype, and whether individuals were infected with a single organism, mixed *C. trachomatis ompA* genotypes, or mixed species (Figure 1). Overall, 28 (35%) of 80 infected individuals demonstrated infection with a single *C. trachomatis ompA* genotype, 16 (20%) were infected with C. psittaci only, eight (10%) with C. pneumoniae only, and 28 (35%) with mixed *C. trachomatis ompA* genotypes and mixed Chlamydiaceae species. Of the single infections, 28 (100%) C. trachomatis, 14 (87.5%) C. psittaci, and seven (87.5%) C. pneumoniae infections were positive for 16S rRNA (by kPCR of cDNA). Twenty-eight (54%) of 52 individuals infected with C. trachomatis had a single infection compared with 16 (53%) of 30 C. psittaci infections and eight (36%) of 22 C. pneumoniae infections; these differences were not statistically significant. Of the mixed infections, 20 (83%) of 24 *C. trachomatis*, 14 (100%) of 14 *C. psittaci*, and 11 (78.6%) of 14 C. pneumoniae infections were positive for 16S rRNA (by kPCR of cDNA). Of the 80 infected individuals, 28 (35%) had infections with C. psittaci, *C. pneumoniae,* or both; single and dual infection with C. psittaci and C. pneumoniae were also strongly associated with a higher odds of developing severe inflammation (OR 4.25 [95% CI 2.9–11.3], *p* = 0.009]. Single infection with C. trachomatis was also strongly associated with severe inflammation (OR 5.7 [95% CI, 3.8–10.1], *p* = 0.002). There was a trend in association of each Chlamydiaceae species with TT but the numbers were small.

**Figure 2:**
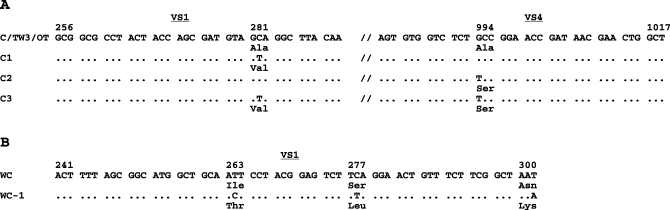
*ompA* Variants of Chlamydiaceae *ompA* Genotypes (A) The sequence-variant regions of *C. trachomatis ompA* genotype C are shown in comparison with reference *ompA* genotype C/TW3/OT. The first sequence section is in variable segment (VS) 1 and the second sequence section after the backward double slash lines is in VS4. The numbers above the sequence regions represent the first nucleotide of VS1, the nucleotide position of the mutation in C1 and C3, the nucleotide position of the mutation in C2, and the penultimate nucleotide of VS4. (B) The sequence variant regions of *C. psittaci ompA* genotype WC-1 are shown in comparison with reference *ompA* genotype WC. The numbers above the sequence regions represent the first nucleotide of VS1 and the three nucleotide positions of the mutations in WC-1.

The single *C. trachomatis ompA* genotype infections (*n* = 28) included: three (30%) of ten B genotypes, 10 (56%) of 18 C genotypes, four (36%) of 11 C1 genotypes, nine (69.2%) of 13 C3 genotypes, and two (67%) of three F genotypes. The remaining 24 C. trachomatis genotype infections were mixed with other genotypes or species and included: 2 Ba genotypes; 2 C2 genotypes; and 1 E genotype*.* None of the B, Ba, E, or F *ompA* genotypes were variants. Figure 2A includes the mutations found in the C. trachomatis C *ompA* genotypes.

Twenty three (82%) of the 28 mixed infections were with mixed species, of which nine were with C. trachomatis and C. psittaci, nine with C. trachomatis and C. pneumoniae, four with both C. psittaci and C. pneumoniae, and one with all three species; five (18%) were mixed *C. trachomatis ompA* genotype infections only (Figure 1). Four of the mixed infections were with two C. trachomatis genotypes and either C. pneumoniae or C. psittaci. There was no evidence of infection with Chlamydia suis, Chlamydophila abortus, Chlamydophila caviae, Chlamydophila pecorum or any other Chlamydiaceae species. Further sequence analysis of the C. psittaci strains in our population revealed high homology with C. psittaci strain WC, a strain that infects the intestine and genital tract of cows. Figure 2B includes the mutations found in the C. psittaci WC *ompA* gene that we termed WC-1.

**Figure 3:**
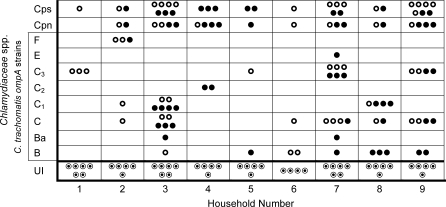
Distribution of *Chlamydia trachomatis ompA* Genotype and Chlamydiaceae Species by Household The vertical axis shows the *C. trachomatis ompA* genotypes and C. psittaci and C. pneumoniae infections represented among the nine households. The designated household number is given on the horizontal axis. The bottom row shows the number of individuals without infections per household denoted by circles containing a dot. Above this row are individuals with infections, denoted as open circles for single infections and filled circles for mixed infections. Since some household members were infected with more than one *ompA* genotype or Chlamydiaceae species, the total number of circles is greater than the number of infected individuals. Cpn, C. pneumoniae; Cps, C. psittaci; CT, C. trachomatis; UI, uninfected.

Figure 3 shows the distribution of *ompA* genotypes and species for members of the nine study households. Each of the three species was detected in eight (89%) study households; one household had no individuals infected with *C. pneumoniae.* When the study individuals were analyzed according to age as a continuous variable or sex, the population did not differ with respect to rates of infection. For the urogenital *ompA* genotypes E and F, E was isolated from an individual 43 y of age in household #7; F was isolated from individuals 3, 5, and 28 y of age in household #2.

Using Chi-square test of significance, we found that individuals who were ≤ 10 y of age were significantly more likely to be infected with C. trachomatis, to have mixed *ompA* genotypes, or to have mixed-species infections. However, they were not more likely than the rest of the population to be infected with C. pneumoniae or C. psittaci (Table 3). There was no association of age with a particular *ompA* genotype or Chlamydiaceae species. Interestingly, *C. pneumoniae and C. psittaci* were evenly distributed throughout all ages.

**Table 3:**
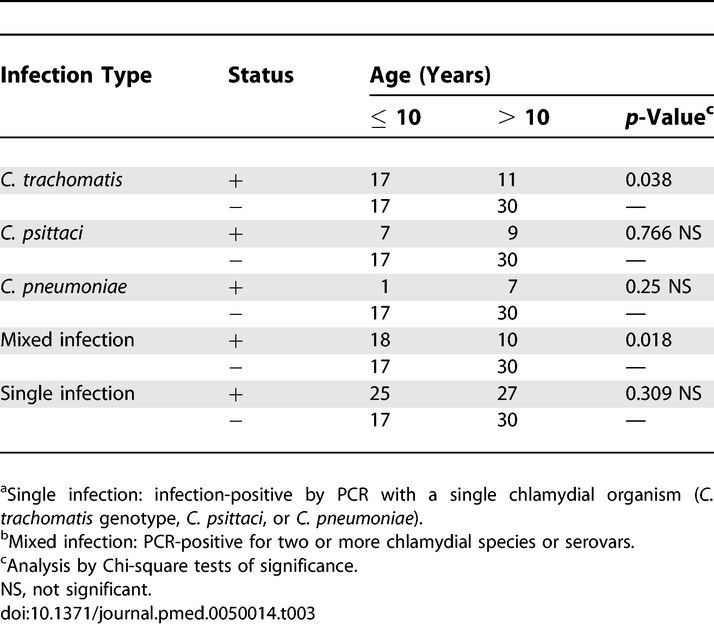
Number of Study Participants with Single^a^ and Mixed^b^ Chlamydiaceae Infections by Age

### Tear and Sera Immunoreactivity to Recombinant Chlamydiaceae Hsp60

Because C. trachomatis Hsp60 is considered an important immunogen in the inflammatory process of chronic infection and has been associated with scarring disease among infected individuals with trachoma [49,50,52,53] and in the monkey model of trachoma [54–56], we were interested in determining the antibody response in tears and sera from our participants who were infected with different Chlamydiaceae species and *ompA* genotypes. One hundred and eight (85%) of 127 individuals were found to have tear IgG antibodies to the three combined recombinant Chlamydiaceae Hsp60s, while 85 (72%) of 118 individuals had serum IgG antibodies to the three combined recombinant Chlamydiaceae Hsp60s. All individuals who were reactive to all three or any one rHsp60 were also reactive to rMOMP, which included all infected individuals. Nineteen (40%) of 47 uninfected individuals had no immunoreactivity to any recombinant Hsp60, and were also not reactive to rMOMP.

There was a significant association between tear and serum IgG immunoreactivity to Chlamydiaceae Hsp60 and evidence for Chlamydiaceae infection as determined by kPCR (Table 4). Pearson Chi-square test was used for significance of the relationship between variables. If the cell number was less than five, a two-sided Fisher exact test was used. Furthermore, a statistically significant association was found for tear and serum immunoreactivity to rCtHsp60 for individuals infected with C. trachomatis (*p* = 0.006 and *p* = 0.023, respectively, by Fisher exact test) and for tear immunoreactivity to rCpsHsp60 for individuals infected with C. psittaci (*p* = 0.013, by Fisher exact test), but not for C. pneumoniae (Table 4)*.* The association between infection and tear immunoreactivity to all three recombinant Chlamydiaceae Hsp60 remained significant both for mixed infections and infection with a single organism, while for serum immunoreactivity, mixed infection was not significantly associated (Table 4).

**Table 4:**
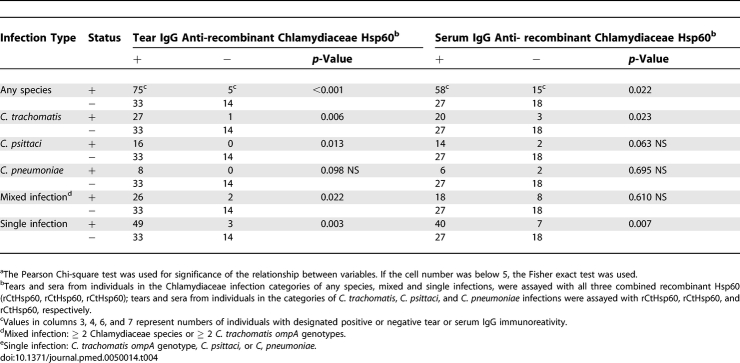
Analysis of Tear and Serum Antibody Response to the Respective Recombinant *C. trachomatis, C. pneumoniae,* and C. psittaci Hsp60 in Trachoma Patients with and without the Respective Chlamydiaceae Infection^a^

By logistic regression controlling for age, sex, and sample clustering method (using the Monte Carlo method), we found that individuals with tear IgG immunoreactivity to the three combined recombinant Chlamydiaceae Hsp60 were eight times more likely than individuals without tear immunoreactivity to be infected (95% CI 6.4–15.1; *p* = 0.003) while individuals with serum IgG immunoreactivity were 4.1 times more likely to be infected (95% CI 3.1–10.1; *p* = 0.014).

There was also a significant association of tear and serum immunoreactivity to rCtHsp60, rCpsHsp60 and the three combined recombinant Chlamydiaceae Hsp60 for individuals infected with C. trachomatis, *C. psittaci,* and all infections, respectively, comparing those with scarring and/or trichiasis to those with no evidence of trachoma (Table 5). Furthermore, there was a significant association of tear and serum immunoreactivity to the three combined recombinant Chlamydiaceae Hsp60 (OR 6.2 [95% CI 4.4–12.6], *p* < 0.001; and OR 3.1 [95% CI 2.1–11], *p* = 0.019, respectively) for individuals with any infection with severe inflammation compared to no trachoma, but not for tear and serum immunoreactivity to individual recombinant Chlamydiaceae species-specific Hsp60. These analyses were performed using logistic regression controlling for age, sex, and sample clustering method. For the latter, we used the Monte Carlo method.

**Table 5:**
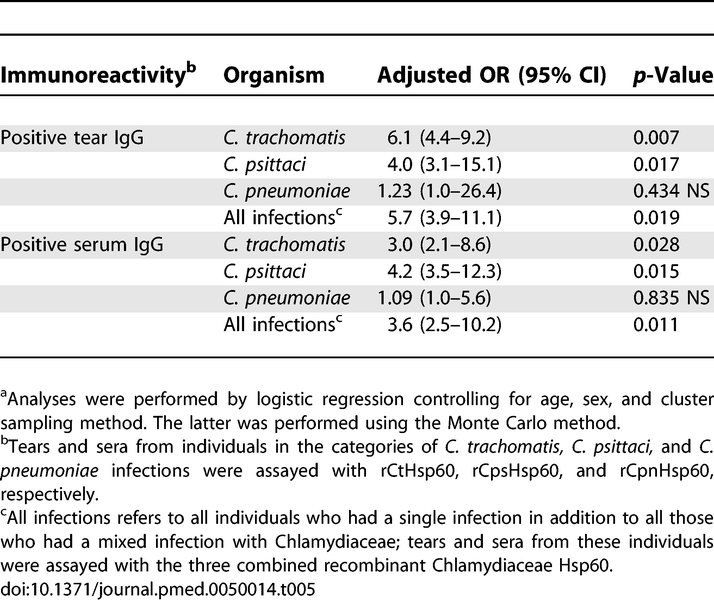
Analyses^a^ for Tear and Serum Immunoreactivity to Chlamydiaceae Species-Specific Hsp60 for Individuals Infected with C. trachomatis, *C. psittaci,* and *C. pneumoniae,* and for All Infections, Comparing Those with Scarring and/or Trichiasis to Those with No Evidence of Trachoma

## Discussion

We report the first evidence that we are aware of for involvement of multiple Chlamydiaceae species in trachoma. The organisms detected from conjunctival samples included C. trachomatis of numerous *ompA* genotypes and two species, C. pneumoniae and C. psittaci, of the *Chlamydophila* genus.

Significantly, non-C. trachomatis organisms accounted for 50% of the ocular infections in this population. All three species were found in all study households, except for one household in which *C. trachomatis ompA* genotypes and C. psittaci were present. Further, 35% of infected individuals had mixed infections, and these consisted primarily of two Chlamydiaceae species. The high agreement between DNA and RNA results also support evidence for viable organisms and not just residual DNA from a previous infection.

Although our data are specific to Nepal, it is likely that C. pneumoniae and C. psittaci are also involved in trachoma in other endemic regions of the world. This hypothesis is supported at least in part by the finding that, in Tanzania, a risk factor for severe trachoma in children is family ownership of cattle [57]. Cattle are an important mammalian host for *C. psittaci,* in which asymptomatic and symptomatic infection and shedding of organisms in intestinal and vaginal secretions is common [47,58]. Furthermore, in Nepal, fresh cow and buffalo dung are used along with mud to construct by hand the walls and floors of huts that are used for living, eating, and grain storage. The fresh dung is also formed into patties that are allowed to dry on the walls of the huts and then used as fuel in fires. These practices present the potential for hand-to-eye transmission of C. psittaci or C. pecorum mammalian strains, although this particular route has not been studied to date. The strains in our study were similar to the bovine WC strain, suggesting that this or related strain types may be important in trachomatous disease in both Asia and Africa.

Identification of C. pneumoniae and C. psittaci in trachoma populations may explain the lack of DNA or culture evidence for C. trachomatis infection among some children and women with obvious clinical signs of follicular, severe, or persistent disease [57,59–63]. Furthermore, additional Chlamydiaceae species may be present in the conjunctiva but not detected because of low copy number and the inability of PCR to amplify limited amounts of DNA. Interestingly, nonhuman mammals such as the Western barred bandicoot (Perameles bougainville) and koala (Phascolarctos cinereus) have also been found to develop ocular conditions similar to trachoma with infections with multiple Chlamydiaceae species, including C. pecorum and C. pneumoniae, a new Chlamydiales endosymbiont of *Acanthamoeba* species, and other uncultured Chlamydiales strains [64,65]. Proof of multiple Chlamydiaceae species involvement in trachoma in other geographic regions will require appropriate studies in Africa, the Middle East, Central and South America, and other regions of Asia where trachoma is endemic.

Although there have been occasional anecdotal reports of laboratory accidents that caused C. pneumoniae and C. psittaci eye infections in humans, C. psittaci is a common ocular pathogen in lower mammalian species [33–35,66]. The observation that vitamin-deficient guinea pigs were susceptible to ocular infection with chlamydiae led to the isolation and identification of a C. psittaci strain (now termed *Chlamydophila caviae*), referred to as the guinea pig inclusion conjunctivitis strain or GPIC [66]. When GPIC organisms were experimentally inoculated onto the eyes of guinea pigs, a delayed hypersensitivity reaction was evident and a self-limited follicular conjunctivitis resulted [35]. Similarly, Chlamydophila felis (formerly C. psittaci, strain Fe/C-56) has been isolated from the eyes of kittens with feline keratoconjunctivitis (FKC). FKC is a follicular conjunctivitis, which, like trachoma, can result in conjunctival scarring and pannus formation [34]. C. pneumoniae has also been isolated from the eyes of Western barred bandicoots [67]. These findings suggest that non-C. trachomatis organisms may be capable of producing the ocular inflammation and scarring sequelae of trachoma.

In studies investigating the natural history of trachoma, the greatest prevalence of infection has been demonstrated in children [1,57,60,62,68,69]. This pattern of infection was supported by the results of this study. Individuals aged 10 y or younger were significantly more likely to be infected with C. trachomatis and with multiple *C. trachomatis ompA* genotypes and species than were adolescents or adults. The role that mixed infections may play in disease pathogenesis has not been explored. It is important to note, however, that children in trachoma-endemic areas commonly develop repeat infections [69], where a history of severe inflammatory disease or persistent infection may be predictors for the subsequent development of scarring and trichiasis [57,68,70–72]. Indeed, in one study by Munoz et al. [73], women aged 18 y and older with conjunctival scars had significantly higher rates of trichiasis and C. trachomatis infection over a 7 y follow-up period compared to women without scars.

The population distribution of C. psittaci and C. pneumoniae infections was more uniform among all age groups and households in this study, perhaps representing a different pattern of transmission and reinfection than for *C. trachomatis*. This distribution pattern suggests that most individuals are susceptible to infection with any of the three species and that these infections are not just sporadic, but widespread in the population.

While the host immune response in trachoma is poorly understood, data suggest that the C. trachomatis Hsp60 may provide an adverse antigenic stimulus [74]. Antibody responses to C. trachomatis Hsp60 have been shown to be associated with a delayed-type hypersensitivity response in the conjunctiva of monkeys [54–56] and guinea pigs [35], and with trachomatous scarring and trichiasis in humans [49,50,52,53], as in this study. A significant association has also been demonstrated between antibodies to C. trachomatis Hsp60 and fallopian tube scarring and infertility in monkeys [75] and women [76,77]**,** which suggests a similar mechanism of disease pathogenesis.

We have previously shown a significant association between elevated tear IgG titers to C. trachomatis Hsp60 and both active and scarring disease among individuals in a trachoma-endemic population in Nepal [49]. While it is possible that the immunoreactivity to recombinant Chlamydiaceae Hsp60 of the different species represents cross-reactivity due to the high degree of homology between species (C. trachomatis Hsp60 is 93% homologous to the Hsp60 of C. psittaci [78] and 85% to that of C. pneumoniae [79]), the associations with scarring and/or trichiasis were significant when assaying tears and sera from individuals infected with one species for responses to the specific recombinant Hsp60 for that species (Table 5). This result suggests that different species elicit a species-specific response. Nonetheless, specific- or cross-reactive antibodies are likely damaging to the conjunctiva; we also found a significant association of tear and serum Chlamydiaceae Hsp60 antibodies with inflammation and scarring for individuals with any infection. These findings may be particularly important since individuals in trachoma-endemic communities are constantly being reinfected and, based on our findings, likely being reinfected with different as well as the same Chlamydiaceae species.

The implication of host involvement in pathogenesis does not rule out the possibility that factors specific to the organism may influence an individual's risk of developing scarring and blindness. One of these factors may be the ability of Chlamydiaceae to persist. Up-regulation of Chlamydiaceae Hsp60 has been demonstrated in an in vitro model of persistence in which aberrant, nonculturable forms of C. pneumoniae and C. trachomatis in HeLa cells were induced by interferon gamma (IFN-γ) [80]. We do not know if persistence occurs in vivo, although supportive evidence is mounting (see review by Hogan et al. [81]). There are some data indicating that C. trachomatis persists in children for months to years in trachoma-endemic settings [57,68,82]. Additional research will be required to determine what role, if any, the different species of Chlamydiaceae play in this process.

If the involvement of multiple Chlamydiaceae species in trachoma is confirmed in other endemic countries, a new approach to antimicrobial therapy may be needed. There has been great interest in the use of azithromycin to treat trachoma, although recurrence of disease and infection is common following cessation of treatment [10,12–14,59,83]. Azithromycin has also been used in the treatment of C. pneumoniae respiratory infections. However, culture-positive persistent infections and elevated minimum inhibitory concentrations following therapy have been reported [84]. In one case series, individuals with evidence for persistent C. pneumoniae pulmonary infections were treated for 10 to 21 d with oral antibiotics, which was not sufficient to resolve infection [85]. In cases of marginal corneal abscesses with follicular conjunctivitis and punctate keratitis due to urogenital C. trachomatis strains, 2 wk of oral antibiotics were required for resolution of clinical disease [25]. There are few data for C. psittaci. Ocular infections with avian strains of C. psittaci required long-term topical and systemic treatment of up to 10 wk for eradication [30,86]. Other reports have described the need for 3 to 10 wk of treatment for ocular infection with non-C. trachomatis species [32,87,88]. The nasopharynx may also be a reservoir for C. trachomatis, *C. pneumoniae,* and possibly C. psittaci [89]. Thus, targeted, systemic treatment with more frequent dosing over a longer time period may be required to prevent persistent infections and host carriage of the organism.

Our study has several limiations. First, our findings are from a single trachoma-endemic region of Nepal, and additional studies will be required to determine whether multiple Chlamydiaceae species are involved in trachoma in other endemic countries of Asia and Africa. It will also be important to determine the number of each species per infection by cloning 16S rRNA and sequencing at least ten randomly selected clones. These data will likely provide further information on the microbial diversity of the conjunctiva that may be involved in active and/or scarring trachoma. In addition, further studies of the host immune response and role of Chlamydiaceae Hsp60 responses in trachomatous disease are warranted. Finally, studies that examine the incidence and prevalence rates of respiratory and urogenital tract infections with the various Chlamydiaceae species would provide a broader context for understanding the role of these organisms in trachoma communities.

In summary, we provide substantial evidence for the involvement of C. psittaci and C. pneumoniae species, in addition to C. trachomatis ocular and urogenital strains*,* in trachoma. The distribution of species by household and age suggests that these infections are widespread. Our data may also explain in part why a proportion of individuals with active trachoma are considered uninfected, since only C. trachomatis-specific diagnostic techniques have been used for detection instead of a broader screen for other Chlamydiaceae organisms. Indeed, 28 (35%) individuals with active trachoma in our study were infected with C. psittaci and/or C. pneumoniae and would have been missed by conventional approaches for detecting C. trachomatis. Furthermore, infection with multiple species may explain the failure of active trachoma cases to resolve their clinical disease following effective C. trachomatis treatment, and the limited effectiveness of the WHO strategy to control trachoma. The high proportion of RNA-positive samples, the significant association of Chlamydiaceae infections with severe inflammation, and the significant association of tear and serum responses to recombinant Chlamydiaceae Hsp60 with inflammation, scarring, and trichiasis for individuals infected with the different species, suggest the importance of all three species in disease pathogenesis. Consequently, our cumulative data suggest that a reevaluation of treatment regimens and approaches to vaccine development may be required. Currently, over 11 genomes of the order Chlamydiales have been sequenced [90–99]. Additional genome sequencing of species and strains responsible for trachoma, together with immunological studies, will inform the design of an efficacious vaccine. Understanding the full impact of Chlamydiaceae species on the epidemiology, immunopathology, and disease outcome of trachoma presents a new challenge for Chlamydiaceae research.

## Supporting Information

### Accession Numbers

The nucleotide sequences for *C. trachomatis ompA* genotypes C1, C2 and C3, and C. psittaci strain WC1 have been deposited at GenBank (http://www.ncbi.nlm.nih.gov/) under accession numbers EU040363, EU040364, EU040365, and EU048338, respectively. The GenBank accession number of *ompA* genotype C/TW3/OT is AF352789, and that of *ompA* genotype WC is AF269269.

## Abbreviations

FKC: feline keratoconjunctivitis
GPIC: guinea pig inclusion conjunctivitis
GST: glutathione-*S*-transferase
kPCR: real-time quantitative PCR
r: recombinant
TF: follicular trachomatous inflammation
TI: intense trachomatous inflammation
T0: absence of trachoma
TS: trachomatous conjunctival scarring
TT: trachomatous trichiasis (inturned eyelashes).
